# Upregulation of Complement Factor H by SOCS-1/3–STAT4 in Lung Cancer

**DOI:** 10.3390/cancers11040471

**Published:** 2019-04-03

**Authors:** Yeon-Hee Yoon, Hyeon-Ji Hwang, Hye-Jin Sung, Sun-Hee Heo, Dong-Sun Kim, Su-Hyung Hong, Kang-Hoon Lee, Je-Yoel Cho

**Affiliations:** 1 Department of Veterinary Biochemistry, The Research Institute for Veterinary Science, College of Veterinary Medicine, Seoul National University, Seoul 08826, Korea; yhyoon999@hanmail.net (Y.-H.Y.); hhyunji1027@naver.com (H.-J.H.); sunghj@protanbio.com (H.-J.S.); jiwoni450@hanmail.net (S.-H.H.); khlee02@snu.ac.kr (K.-H.L.); 2 Department of Oral Microbiology, School of Dentistry, Kyungpook National University, Daegu 41566, Korea; hongsu@knu.ac.kr; 3 Department of Anatomy, School of Medicine, Kyungpook National University, Daegu 41566, Korea; doskim@knu.ac.kr

**Keywords:** complement factor H, CFH, STAT4, SOCS, lung cancer

## Abstract

Complement factor H (CFH) is a fluid phase regulator of complement proteins and functions to prevent complement attack and immune surveillance. CFH is known to inactivate therapeutic antibody-dependent complement-mediated cellular cytotoxicity. We found that CFH was highly expressed in human lung cancer cells and tissues. To investigate mechanisms of CFH upregulation, we searched for a CFH transcription factor and its regulatory factors. First, signal transducer and activator of transcription 4 (STAT4) expression patterns coincided with CFH expression patterns in lung cancer tissues. Knockdown of STAT4 led to decreased CFH secretion from lung cancer cells. STAT4 bound directly to the CFH promoter, as demonstrated by luciferase reporter assay, electrophoretic mobility shift assay (EMSA), and chromatin immunoprecipitation (ChIP) assay, suggesting that STAT4 is a transcription factor for CFH. In addition, a low level of suppressors of cytokine signaling (SOCS)-1/3, a Janus kinase (JAK) inhibitor, was observed in lung cancer cells and its transfection decreased CFH protein levels and promoter activity. Unexpectedly, the low level of SOCS-1/3 was not due to epigenetic silencing. Instead, differential methylation was found on the regulatory region of STAT4 between normal and lung cancer cells. In conclusion, our results demonstrated that CFH is upregulated by constitutive activation of STAT4, which is accounted for by SOCS silencing in lung cancer cells.

## 1. Introduction

Lung cancer accounts for the highest mortality rate among all cancers [1,2]. This is, in part, due to undiagnosed subjective symptoms at the early stages of cancer and common metastasis to other organs after diagnosis [2,3]. To improve the poor survival rate and avoid metastasis to other organs, new approaches are needed for early diagnosis and effective cancer treatment. 

Recent advances in understanding the biology and molecular basis of lung cancer have demonstrated that the application of monoclonal antibodies (mAbs) against tumor-associated antigens can elicit complement-mediated tumor cell lysis [1,3,4]. In fact, rituximab (for the treatment of non-Hodgkin’s lymphomas, mAb anti-CD20), cetuximab (mAb anti-EGFR), and bevacizmab (mAb anti-VEGF-A) have been widely used as cancer therapy drugs [5,6,7]. These mAbs enhance complement activation and evoke antibody-dependent cellular cytotoxicity (ADCC) and apoptosis [3,5]. Despite these two potent mechanisms, the therapeutic effect of mAbs is incomplete [3,4,5]. The incomplete effects of mAbs on tumor cells are partially due to the existence of protective complement inhibitory mechanisms that interfere with mAb and Complement 3 (C3) interactions with cancer cells [3,8]. 

The complement system can be activated by three different pathways: classical, alternative, and mannose-binding lectin pathways which activate C3 fragments. Activated C3 triggers the lytic pathway that forms the end product, membrane attack complex (MAC), to induce target cell destruction. The cascade mechanism of complement proteins is highly regulated at each stage by membrane-bound and fluid phase regulators. C4BP is known to be a major fluid phase regulator of the classical pathway, while complement factor H (CFH) and its spliced form, factor-H-like protein 1 (FHL-1), are considered to be the main fluid phase regulators of the amplification loop/alternative pathway of complement activation. Both of these proteins inhibit the activation of the complement system and thus can protect host cells [4,9,10,11]. 

FH is a 155-kDa sialic acid-rich glycoprotein found in human plasma and is constitutively produced by the liver and other cell types, including endothelial and epithelial cells [1,12,13]. The principle role of CFH is to inactivate the C3 convertases in the alternative complement system pathway, by either competitively dissociating Bb from the C3bBb complex or acting as a cofactor for the factor-I-mediated cleavage of C3b [11]. 

Defects or deficiencies in CFH caused by genetic variation, infection, and oxidative stress provoke chronic inflammation and tissue injury, which can result in various diseases of the kidneys, eyes, and brain [14,15,16]. Additionally, several pathogenic microorganisms escape the complement attack either by mimicking FH or by recruiting host FHs to their surfaces by using FH-binding proteins. In that case, FH inhibits C3 participation in the complement system and promotes the pathogenicity of the microorganism [17,18]. 

FH is also secreted in several cancers, such as nonsmall cell lung cancer, ovarian cancer, and colon cancer [1,3,4]. Cui et al. reported FH overexpression in human lung cancer tissues (53%) in their immunohistochemistry analyses, and that adenocarcinoma patients with highly expressed CFH have a shorter survival time [19]. Importantly, lung cancer cells demonstrate a high level of resistance to complement-mediated lysis via activation of the classical pathway, as well as via the alternative pathway due to the role of CFH and FHL-1 [20]. Thus, the inhibition of CFH in cancer cells may improve anticancer therapy. Although many reports have demonstrated CFH’s expression, functions, and complex interactions with other regulatory proteins in the complement system, no information is known about the molecular mechanisms of CFH expression in cancers. In this study, we investigated the signaling mechanism that elicits CFH expression in lung cancer.

## 2. Results

### 2.1. CFH Expression in Lung Cell Lines, Human Lung Tissues, and Primary Cultured Lung Cells

**Figure 1:**
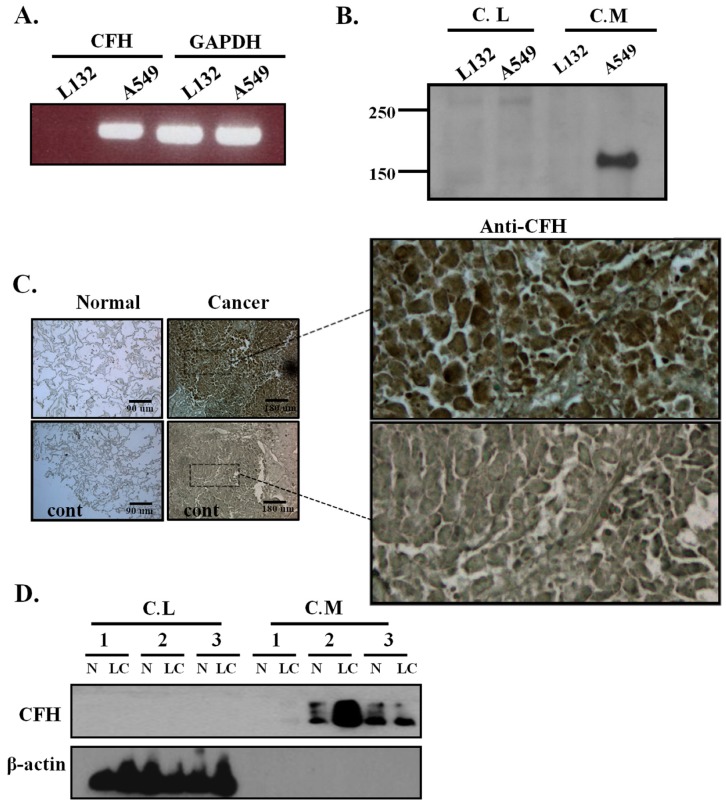
Expression of complement factor H (CFH) in lung cell lines and human lung tissues. (**A**) The expression level of CFH was examined by RT-PCR in L132 and A549 cells. Glyceraldehyde-3-phosphate dehydrogenase (GAPDH) was used as a control. (**B**) FH immunoblot analysis in the conditioned media and cell lysates of L132 and A549 cells. After harvesting, the serum-free conditioned media were filtered through pore size 0.2 µm and then precipitated with TCA and acetone. The precipitated media and cell lysates were prepared with RIPA buffer containing protease inhibitors and were used for immunoblot analysis. (**C**) Representative immunohistochemistry of FH expression in human lung tissues. The figure shows an example of tumors with different levels of FH expression compared with normal controls. Scale bar = 90 µm in normal and 180 µm in cancer. Selected regions were enlarged. (**D**) FH Western blot analysis of cell lysates and conditioned media from human lung tissue primary cultures. The FH expression was higher in the conditioned media of the primary lung cancer cells than in the primary normal lung cells (patients #1 and #2). N: normal, LC: lung cancer. The number represents the patient.

The expression level of CFH was measured in lung cell lines by RT-PCR. CFH mRNA expression was detected at high levels in A549 lung cancer cells but was not detected in the L132 normal lung epithelial cell line (Figure 1A). The mRNA expression of other complement regulators—CR1, CD46, CD55, CD59, as well as FHL-1—were also tested in these cell lines (Supplementary Figure S1). Compared to the L132 cell line, A549 had a higher expression of FH/FHL-1, CD35, and CD59 but a lower expression of CD46 and CD55. To test whether FH is secreted into the culture medium, cells were starved for 48 h, and then the conditioned media and cell lysates were tested for FH by Western blot analysis. A high level of FH expression was detected in the conditioned medium of A549 cells but not in the conditioned medium from L132 cells (Figure 1B). To investigate whether the higher FH expression level in lung cancer cell lines are translated to primary cells, we performed immunohistochemistry staining on human lung tissues and Western blot analysis on the primary culture of human lung tissues. The immunohistochemical stain showed a high level of FH expression in human lung cancer tissues compared with normal lung tissues (Figure 1C). In the primary culture of human lung tissues, we observed that FH levels were also high in two out of three conditioned media of lung cancer cells compared with that of primary normal lung cells (Figure 1D). In both the cell lines and primary cell cultures, we could not detect FH in the cell lysates (Figure 1B,D). These results suggest that the increased level of FH in lung cancer is not limited to lung cancer cell lines but also exists in human lung cancer tissues and primary cultured lung cancer cells.

### 2.2. CFH Expression Levels Correlate with STAT4 Expression Levels in Lung Cancers 

**Figure 2:**
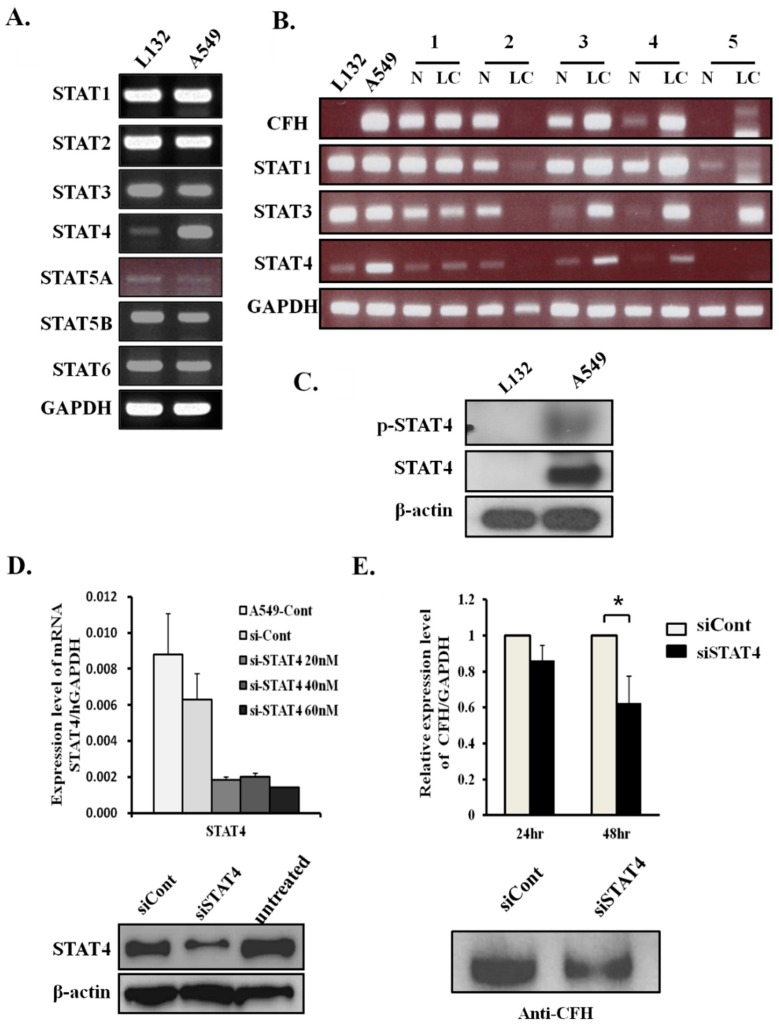
CFH expression level is regulated by STAT4. (**A**) The mRNA expression analysis of the STAT family by RT-PCR in L132 and A549 cells. (**B**) RT-PCR analysis of STAT1, STAT3, STAT4 and CFH in human lung cell lines and lung tissues. The number represents the patient. N: adjacent normal tissue, LC: lung cancer tissue. (**C**) The p-STAT4 expression level in lung cell lines. Cell lysates were extracted with RIPA buffer containing 1 mM Na_3_VO_4_, 5 mM NaF, and complete protease inhibitors. Total cell lysates were immunoblotted with an antibody against STAT4 and p-STAT4 (Tyr693). β-Actin was used for the loading control. (**D**) A549 cells were transfected with siCont and siSTAT4. To determine the optimal concentration of siRNA, various concentrations of siSTAT4 (0, 20, 40, and 60 nM) were applied before real-time RT-PCR (upper panel). Western blot analysis was performed in A549 cells treated with siRNA 60 nM for 48 h (bottom panel). (**E**) A549 cells were transfected with 60 nM of control (siCont) or 60 nM of siSTAT4 for 48 h, and CFH levels were analyzed by real-time RT-PCR (upper panel) and Western blot analysis (bottom panel) in serum-free conditioned media. The bars represent the mean ± SD calculated from three independent experiments done in triplicate (* *p* < 0.05 by Mann–Whitney *U* test).

In general, when epithelial cells are treated with interferon gamma (IFN-γ), FH secretion is increased [21,22]. Although there are no stimuli, lung cancer cell lines constitutively express high levels of FH. To identify relevant transcription factors for CFH in lung cancers, the mRNA expression levels of the STATs were tested in both L132 and A549 cells (Figure 2A). STAT4 level was higher in A549 cells than in L132 cells, whereas the levels of the other STATs were similar or a little higher in the L132 cells. Next, to investigate any correlation between CFH expression and the expression of STATs in human normal lung tissues and lung cancer tissues, the transcription level of CFH was compared with STAT1, STAT3, and STAT4. As shown in Figure 2A, STAT4 was higher in the A549 cell line than the L132 cell line. STAT1 and STAT3 were also tested because STAT1 was reported to be a transcription factor for CFH in response to the stimulation of IFN-γ [22], and STAT3 is an oncogene that is also reported to be increased in lung cancers [23].

Our results showed STAT1 levels were higher in cancer tissues #3 and #4S, while STAT3 levels were higher in cancer tissues #3, #4, and #5, but the correlation with CFH expression was weaker than that of STAT4. Our results showed that CFH expression levels were well correlated with STAT4 expression levels (Figure 2B). In A549 cells and cancer tissues #3 and #4, STAT4 expression was higher in the tissues that also had a high expression of CFH. In addition, in the paired tissues where STAT4 expression levels were similar between normal and cancer cell lines, the CFH levels were also similar (tissue #1). When STAT4 was not expressed, CFH was not expressed (tissue #5). Therefore, these results suggest that the expression of STAT4 might be related to CFH expression and have a potential role as a transcription factor for CFH in lung cancers. 

### 2.3. STAT4 Regulates CFH Expression Levels

The protein expression level of STAT4 was also high in the cell lysates of A549 cells but not in lysates from L132 cells (Figure 2C, second panel). STATs are activated by tyrosine phosphorylation, which is generally temporary and strictly modulated. However, several persistently activated STATs have been observed in many cancer cells [23]. To test whether STAT4 exists in the activated form in A549 cells, p-STAT4 antibodies were used for Western blot analysis in total cell lysates. Activated p-STAT4 was detected in A549 cells but not in L132 cells (Figure 2C, first panel).

To evaluate the effect of STAT4 knockdown on the expression of CFH in A549 cells, siRNA was introduced into the cells. To find an optimal siRNA concentration for the knockdown of STAT4, we treated the cells with siSTAT4 at concentrations of 20, 40, and 60 nM and then performed real-time RT-PCR and Western blot analysis on A549 cells (Figure 2D). STAT4 levels were 77% lower in cells treated with 60 nM of siSTAT4 than in siCont-treated cells (Figure 2D, upper panel). The STAT4 protein level also dramatically decreased with 60 nM of siSTAT4 (Figure 2D, bottom panel). Additionally, we found that the mRNA and protein levels of CFH were significantly decreased by 60 nM of siSTAT4 in A549 cells (Figure 2E). These results suggest that the STAT4 transcription factor regulates CFH expression in A549 cells.

### 2.4. STAT4 Upregulates the Activity of the CFH Promoter 

**Figure 3:**
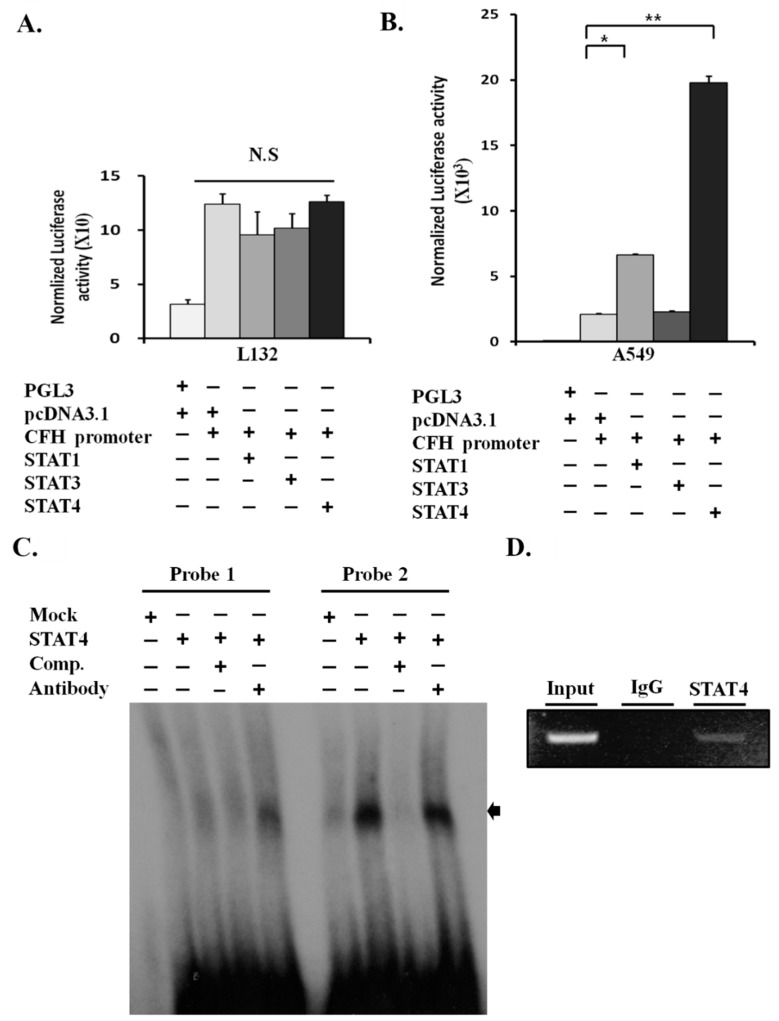
STAT4 regulates CFH expression as a transcription factor. (**A**,**B**) The promoter activity of CFH was examined in L132 and A549 cells transfected with CFH luciferase reporter constructs and STATs. Two days after transfection, luciferase activities were measured. The bars represent the mean ± SD calculated from three independent experiments done in triplicate (* *p* < 0.05; ** *p* < 0.01 by Mann–Whitney *U* test). (**C**) The electrophoretic mobility shift assay (EMSA) showed specific binding of the STAT4 protein to the probe 2 position. The 32P-labeled probe 1 (−950 to −927-nt region) and probe 2 (+161 to +184-nt region) were incubated with the STAT4 protein. To verify the band identity as a STAT4–DNA complex, cold competitor (comp.) with 100-fold molar excess of unlabeled probe and anti-HA antibody (Santa Cruz) for the supershift assay were added before the probe incubation. Mock: pcDNA3.1 plasmid DNA added. (**D**) Chromatin immunoprecipitation (ChIP) assays for the analysis of endogenous STAT4 binding to the region containing probe 2 in the CFH promoter. ChIP was done using anti-STAT4 or control IgG [22].

To test whether STAT4 is a direct transcription factor for CFH, we examined the CFH promoter activity by luciferase assay in L132 and A549 cells. First, cells were transiently transfected with STAT1/3/4 cDNAs and CFH promoter constructs without any stimulation (Figure 3A,B). The CFH promoter activity was not changed by STAT1, STAT3, or STAT4 transfection in L132 cells (Figure 3A). It is of note that endogenous STAT4 proteins and pSTAT4 were not detectable in L132 cells (Figure 2C). The fact that transfection of exogenous STAT4 alone did not result in the activation of CFH promoter activity suggests that L132 cells may not have p-STAT4 activating signals or key STAT4 cofactors that act together on the CFH promoter. There was also no difference in CFH luciferase activity between groups, and the CFH promoter activity levels were generally very low in L132 cells (Figure 3A). In contrast, the luciferase activity of the CFH promoter was remarkably increased in the A549 cells transfected with STAT4 compared with the mock control (Figure 3B). The increase in luciferase activity was more significant in cells transfected with STAT4 than with STAT1, and STAT3 did not induce any CFH promoter activity. 

### 2.5. STAT4 Directly Binds and Activates the CFH Promoter 

We have shown that STAT4 upregulates CFH mRNA and protein levels (Figure 2D,E) and increases its promoter activity (Figure 3B). Next, to test whether the STAT4 protein binds to the CFH DNA promoter region, electrophoretic mobility shift assay (EMSA) analysis was performed. STAT binding sites on the CFH promoter were analyzed using TFSEARCH, a transcription factor search engine (PAPIA system). 

Two putative STAT binding sites were detected on the 1.2-kb CFH promoter sequence. These two potential STAT binding sites were between −950 and −927 nt (EMSA Probe 1) and +161 and +184 nt (EMSA Probe 2). STAT4 formed a strong complex with radiolabeled probe 2 and the band disappeared via cold competitor (Figure 3C). Although the band representing the STAT4–probe 2 complex did not produce a clear supershift after the addition of anti-HA antibody, the band intensity was decreased. Our results revealed that the probe 2 region (+161 to +184 nt) is potentially a STAT4 binding site.

To further confirm whether endogenous STAT4 directly binds to the probe 2 region of the CFH promoter, a chromatin immunoprecipitation (ChIP) assay was performed using an anti-STAT4 antibody [22]. The 521-bp PCR product of the CFH promoter demonstrated that STAT4 was bound to the endogenous CFH promoter in the chromatin of A549 cells (Figure 3D). Together, these results demonstrated that STAT4 directly binds to the CFH promoter as a transcription factor and activates CFH expression. 

### 2.6. Suppressor of Cytokine Signaling (SOCS) Proteins Negatively Regulate CFH Expression by Suppressing STAT4 Activity

**Figure 4:**
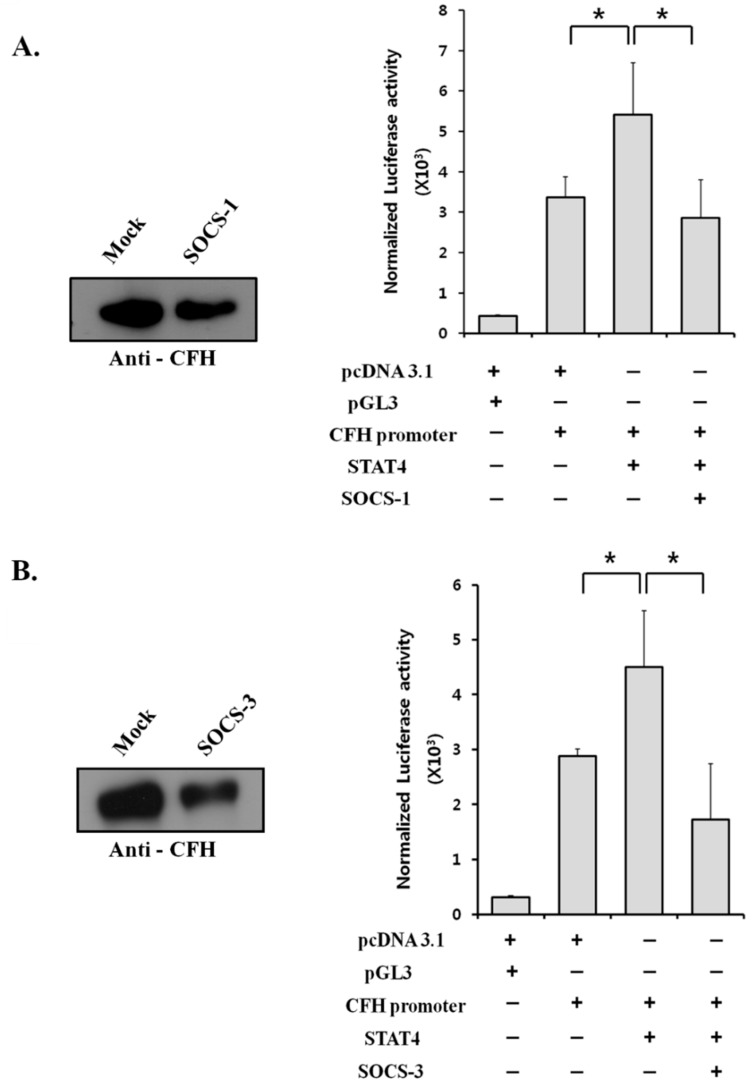
The effects of suppressors of cytokine signaling (SOCS) on the STAT4 activity and the CFH expression in A549 cells. (**A**,**B**) The protein expression of CFH in the conditioned media of the transfected A549 cells was analyzed after the transfection of SOCS-1 (**A**, left) or SOCS-3 (**B**, left). The promoter activity of CFH was examined in the A549 cells transfected with STAT4 and SOCS-1 (**A**, right) or SOCS-3 (**B**, right). The bars represent the mean. ± SD calculated from three independent experiments done in triplicate (* *p* < 0.05 by Mann–Whitney *U* test).

We have shown that STAT4 is responsible for the upregulation of CFH in lung cancer cells. The factors that affect CFH overexpression are usually cytokines that regulate the STAT pathways [22]. Therefore, we hypothesized that overexpression of CFH in lung cancer is the result of upstream factors that are involved in cytokine signaling, dysregulating the activity of STAT4. STATs are regulated at many points of the signaling pathway and the SOCS proteins are known negative regulators of STATs during cytokine signaling [24]. STAT4 is phosphorylated by JAKs, which are negatively regulated by SOCS-1 and SOCS-3 [25,26,27]. To investigate whether SOCS-1 and SOCS-3 regulate STAT4 activity, an upstream component of CFH expression, SOCS-1 and SOCS-3 plasmids were transfected into A549 cells. FH expression was decreased in the conditioned media of the A549 cells transfected with SOCS-1 or SOCS-3 plasmids (Figure 4). In the luciferase assay, A549 cells transfected with SOCS-1 and SOCS-3 along with STAT4 showed significantly lower CFH promoter activity than the cells transfected with STAT4 alone (Figure 4A,B). These results suggest that SOCS-1 and SOCS-3 negatively regulate STAT4 activity. 

### 2.7. Epigenetic Regulation of SOCS-1, SOCS-3, and STAT4 Expression in Lung Cancer Cells 

**Figure 5:**
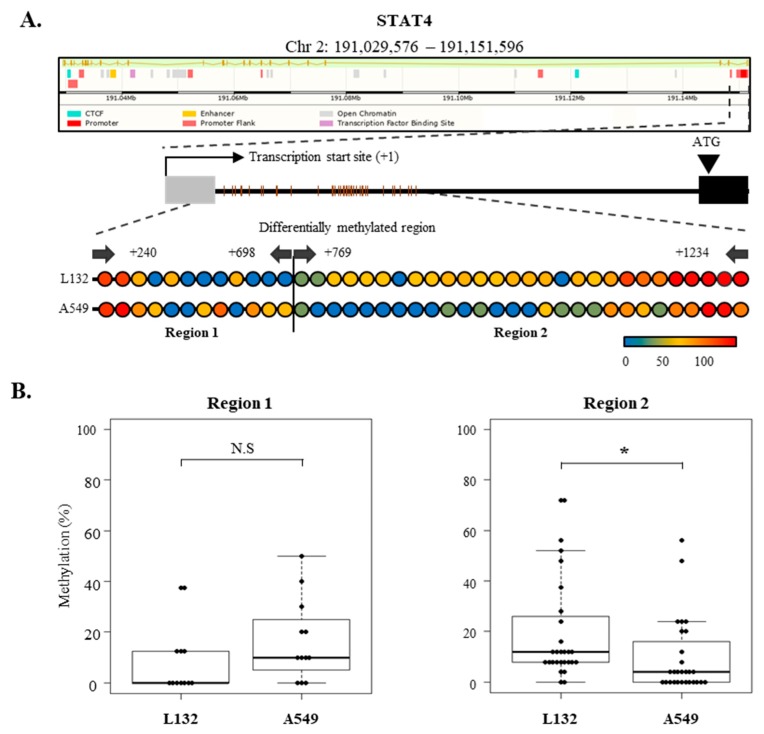
Differential methylation on the STAT4 regulatory region in lung cancer cells. (**A**) Partial structure of the 5′ genomic region of the STAT4 gene was obtained from the Ensembl dog genome browser. Targeted CpGs are depicted with genetic features, TSS, exon, and intron. Arrows indicate PCR primers. The percentage methylation on each CpG site was calculated and depicted in a lollipop plot with a color scale. (**B**) Overall methylation is presented as a boxplot and statistical significance was measured by * *p* < 0.05, Student’s *t*-test.

**Figure 6:**
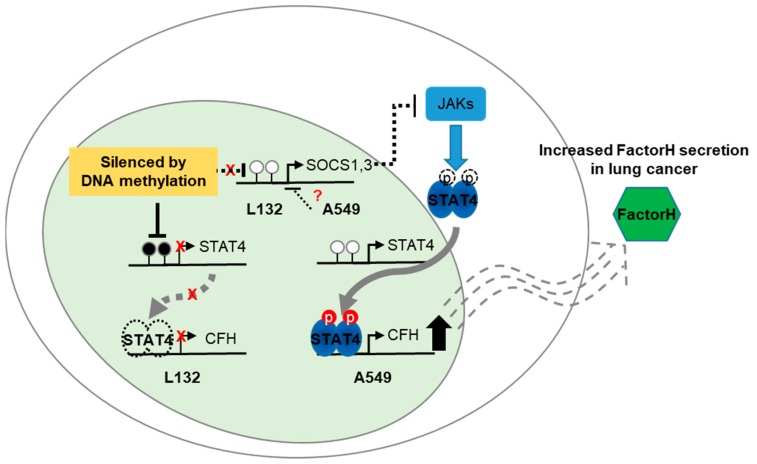
Schematic diagram for a proposed molecular mechanism of CFH regulation in lung cancers. STATs are phosphorylated by JAKs and other tyrosine kinases. Phosphorylated STATs translocate to the nucleus and drive transcription of target genes. Activated STATs are negatively regulated by SOCS family proteins, phosphatases, and PIAS proteins. The current study demonstrated that constitutively phosphorylated STAT4 directly binds to the CFH promoter and drives the transcription of CFH. However, for some reason, such as epigenetic modification excluding promoter methylation, the expression of SOCS was low in lung cancer, resulting in no negative feedback of the JAK/STAT pathway, causing the overexpression of CFH. The promoter regions of the SOCS family were unmethylated in both normal and lung cancer cells, indicating that methylation is not involved in differential regulation of SOCS family expression in lung cancer. Instead, the methylation status was significantly different in the regulatory region of STAT4; as a result, CFH is overexpressed. Consequently, CFH overexpression is due to the regulatory imbalance in the JAK–STAT–SOCS signaling pathway. In particular, the action of constitutively phosphorylated STAT4 and silenced SOCS family plays an important role in lung cancer.

We accordingly investigated whether epigenetic controls, such as promoter methylation, are involved in the regulation of the SOCS family and STAT4 activity. The SOCS family proteins are known to be silenced in various cancers [28,29,30,31]. We thus analyzed methylation levels on the regulatory regions of the SOCS family and STAT4 in A549 and L132 cells (Figure 5A). Unexpectedly, there was no difference in methylation levels between the promoter regions of SOCS-1 and SOCS-3; both were demethylated in A549 and L132 cells, which means that the activity of the SOCS family might not be regulated by promoter methylation in the A549 lung cancer cell line (Supplementary Figure S2). On the other hand, STAT4 methylation on the regulatory region in A549 cells was significantly distinct from that in L132 cells (Figure 5B). Shin et al. reported differential methylated regions of which the methylation showed an anticorrelation with STAT4 expression in a T-cell-specific manner [32]. In the present study, lower methylation was found in A549 lung cancer cells than in L132 normal cell lines, which is reversely consistent with higher STAT4 RNA expression in A549 than in L132 cells (Figure 2). Altogether, this result revealed that CFH overexpression in A549 cells and lung cancer tissue is under the control of STAT4 activation, which is known to be mediated by the JAK and SOCS cytokine signaling pathway. Additionally, DNA methylation was involved in this regulation through the differential regulation of STAT4 and not of the SOCS family in L132 and A549 cells (Figure 6). 

## 3. Discussion

CFH is overexpressed in cancers, which allows cancer cells to avoid complement-mediated attack [1,3]. There has been no report that describes the molecular mechanisms of CFH overexpression in lung cancers. Here, we studied the factors that influence CFH overexpression and their related mechanisms in lung cancer. The major findings of the present study can be summarized as follows: (1) CFH and STAT4 expression levels were correlated in lung cell lines and human lung cancer tissues; (2) STAT4 regulated CFH expression by directly binding the CFH promoter as a transcription factor; (3) the SOCS family negatively regulated CFH expression by suppressing the STAT4 pathway; and (4) epigenetic gene regulation, such as methylation, did not influence the expression of the SOCS family but did regulate STAT4 expression in lung cancer. Hypomethylation of the STAT4 regulatory region can activate the JAK/STAT4 pathway, resulting in CFH overexpression in lung cancers (Figure 6).

A few studies have reported FH expression in various tumor cells. Cui et al., reporting CFH expression in a tumor-cell-type-dependent manner, observed that non-small cell lung cancer cell lines but not small cell lung cancer expressed FH, and that high FH expression was significantly correlated with lung adenocarcinomas with worse prognoses [19]. Additionally, it was reported that tumor cells expressing CFH on their surface can prevent C3b accumulation on their cell membranes and increase the resistance of these cells to complement-mediated lysis [33]. Previous reports have suggested that CFH expression levels were increased as a result of IFN-γ stimulation in epithelial, endothelial, and other cell types [13], and STAT1 was reported to be a transcription factor for CFH [22]. Regarding the regulation of CFH, other studies have revealed that miRNA-146a is highly complementary to the 3′-UTR region of CFH, and upregulation of miRNA-146a by NF-κB negatively regulates CFH expression. CFH was also decreased by IL-1β, Aβ42, and oxidative stress in human neural cells [15]. Similar to NF-κB, IL-1β and oxidative stress are usually prominent in cancer cells, but the upregulation of miRNA-146a by these factors cannot explain the high expression of CFH in lung cancers. Depending on the cytokine stimuli, the known transcription factors that regulate expression of CFH are NF-κB and STAT1 [15,22]. A discrepancy was observed between a previous study, which stated that STAT1 is a transcription factor that mediates the activation of CFH in retinal pigment epithelial cells stimulated with IFN-γ [22], and our current results showing that CFH is more significantly affected by STAT4 than by STAT1 in A549 cells. We demonstrated that CFH expression levels are highly correlated with STAT4 expression levels in human lung cell lines and human lung cancer tissues without any stimuli. CFH expression was detected in three out of five adjacent normal lung and cancer tissues where STATs are expressed. However, CFH was not amplified in two normal and one cancer tissues that did not express STATs. This might indicate that STATs are a key regulator of CFH expression in human lung cancer. We also showed that phosphorylated STAT4 and CFH expression were decreased by STAT4 knockdown, indicating that CFH expression levels are regulated by STAT4 in lung cancer. Thus, STAT4 activation causes CFH overexpression that allows lung cancer cells to escape from complement-mediated immune system attack. 

STAT4 is highly expressed in lymphoid and myeloid tissues and testis, but it is relatively restricted in other tissues. However, more recent investigations suggest that STAT4 might also be expressed in other tissues, and this protein is known to have various functions in nonimmune cells [34,35]. Recent reports suggested that genetic variations in STAT4 in rectal cancer are associated with rectal cancer survival [36]. However, the pathogenicity mechanism of STAT4 in carcinogenesis remains poorly understood. One revealed mechanism is that STAT4 can activate the S100A4 promoter by interacting with HBXIP, which increases the expression of S100A4 and enhances the proliferation and migration of breast cancer cells [37]. Our study demonstrated that STAT4 stimulates CFH upregulation in lung cancers. The high level of FH may provide the lung cancer cells a means to escape immune surveillance and maintain their stability. The EMSA and CHIP assays showed that STAT4 regulates CFH expression by binding directly on the CFH promoter as a transcription factor. The STAT4 binding site was previously shown to be a STAT1 binding site on the CFH promoter after treatment with IFN-γ [22]. The constitutive activation of STAT proteins is commonly detected in diverse human cancer cell lines and a wide variety of solid tumors, which suggests that abnormal JAK–STAT signaling contributes to malignant transformation by controlling cell growth, survival, proliferation, and angiogenesis [23,26,28]. 

JAK–STAT signaling is regulated by various proteins, including SOCS proteins, protein inhibitor of activated STAT (PIAS), and protein tyrosine phosphatases, through distinct mechanisms [24]. SOCS-1 binds to JAKs, directly inhibiting JAK activation, and also regulates several other cytokines and hormone receptor systems, including EGFR signaling [24]. SOCS-1 is also a key regulator of IFN-γ and the IL-12-activated signaling pathway [25]. SOCS-3 suppresses JAKs by directly binding to cytokine receptors [24]. Thus, SOCS-3 can regulate the signaling of several cytokines and inhibits STAT4 [27]. Together, SOCS-1 and SOCS-3 play important roles as tumor suppressors by negatively regulating the activation of STATs [31,38]. Our results showed the presence of phosphorylated STAT4 in lung cancer cells (Figure 2C). The STAT4 activity on the CFH promoter was significantly suppressed by SOCS-1 and SOCS-3 (Figure 4). SOCS proteins inhibit JAKs and cytokine receptors, leading to decreased activity of STAT4. The decrease in STAT4 activity results in the decreased expression of CFH. Together, we concluded that SOCS-1 and SOCS-3 negatively regulate CFH expression by inhibiting JAKs and STAT4. 

Concomitantly, we investigated epigenetic regulation of the gene expression of SOCS-1 and SOCS-3 as well as STAT4 in A549 and L132 cells (Figure 5). However, there was no difference between the promoter methylation level of SOCS-1 and SOCS-3 in both A549 and L132 cells; both were unmethylated in both cell lines (Supplementary Figure S2), suggesting that aberration of promoter methylation of the SOCS family was not involved in its gene-expression decrease in A549 cancer cells. It seems that there is a discrepancy between this result and previous studies reporting that SOCS-1 and SOCS-3 are silenced by hypermethylation of the gene in tumors. It is supposedly caused by a difference in the analyzed cell types and promoter regions. The reverse correlation found between hypermethylation and silenced SOCS-3 was tested in the second exon (gene body), not in the promoter region, and hypermethylation of SOCS-1 promoter was found in certain hepatocellular carcinoma cell lines [30,31]. It can be speculated that the CFH promoter is silenced by promoter methylation and histone modification in L132 cells. However, treating L132 cells with 5-Aza-2’-deoxycitidine (5-AzadC) did not induce SOCSs expression (Supplementary Figure S2), suggesting that epigenetic silencing might not be the direct mechanism responsible for the nonexpression of CFH in L132 cells. 

## 4. Materials and Methods 

### 4.1. Lung Cell Lines and Human Lung Tissues 

L132, a normal lung cell line, and A549, a lung adenocarcinoma cell line, were obtained from the ATCC (Manassas, VA, USA) and were cultured in DMEM and RPMI 1640 (GIBCO, Gran Island, NY, USA), respectively, with 10% FBS (GIBCO). Human lung tissues were received from Kyungpook National University Hospital (Daegu, South Korea) and were used for molecular analysis and primary culture. Fresh human lung tissues were minced by scissors, then treated with collagenase type I (0.5 mg/mL, Worthington, NJ, USA), incubated at 37 °C for 1 h, centrifuged, and seeded on plates; they were maintained with 10% FBS supplemented DMEM. All cells were incubated at 37 °C in a humidified atmosphere containing 5% CO_2_.

### 4.2. Antibodies and Western Blot Analysis

Western blot analysis was performed as previously described [2]. The antibodies used were monoclonal anti-human FH (34F12 (LF-MA0133), AB Frontier, Seoul, South Korea), phospho-STAT4 (p-STAT4, Tyr693, R&D Systems, Minneapolis, MN, USA), β-Actin (Sigma-Aldrich, St. Louis, MO, USA), and STAT4 (Cell Signaling, Beverly, MA, USA). Anti-mouse IgG and rabbit IgG (Cell Signaling) were used as secondary antibodies. 

### 4.3. RNA Extraction and RT-PCR

RNA extraction and RT-PCR were performed as previously described [39]. Total RNA was extracted from the cells and tissues using TRIzol reagent (Invitrogen, Carlsbad, CA, USA) according to the manufacturer’s instructions. PCR primers for the target genes are listed in Supplementary Table S1. 

### 4.4. Immunohistochemistry 

Immunohistochemistry was performed as previously described [40]. Following the procedure, CFH antibody at a dilution of 1:100 was added to human lung tissues.

### 4.5. DNA Constructs

The CFH promoter luciferase reporter construct was generated by PCR amplification of the −1061 to +185-nt region around the CFH transcription start site using human genomic DNA (Takara, Shuzo, Japan) as the template. The PCR products were inserted into the NheI and XhoI sites of the pGL3 Basic vector (Promega, Madison, WI, USA). STAT4-HA constructs were also produced by PCR amplification using STAT4 in pBluescript (Open Biosystems) as the template. The PCR products were cloned into the EcoRI and XhoI sites of pcDNA3.1 (Invitrogen, USA). All restriction enzymes were acquired from New England Biolabs (Beverly, MA, USA). SOCS-1 and -3 plasmids were kindly provided by Dr. Albert Duschl and Dr. Jutta Horejs-Hoeck (University of Salzburg). STAT1 construct was obtained from Dr. David A. Frank (Harvard Medical school) and STAT3 from Dr. Joo-Yeon Yoo (POSTECH)

### 4.6. Luciferase Assay

Luciferase assays were performed as previously reported [39]. Briefly, cells were transfected using the Neon transfection system (for 30 ms, 2 pulses at 1230 V; Invitrogen, Canada) according to the manufacturer’s instructions. After 48 h, cell lysates were used in the luciferase assay system (Promega).

### 4.7. RNA Interference Knockdown

The oligonucleotides used for the STAT4 siRNA and scrambled control siRNA (Thermo Scientific, USA) were transfected into cells using Dharmaffectine (Thermo scientific) according to the manufacturer’s instructions. Transfected cells were grown in serum-free media for 48 h. After this incubation, conditioned media and cell lysates were used for CFH detection and analyzed for STAT4 expression.

### 4.8. EMSA 

EMSAs were done as previously described [39]. Double-stranded DNA probes for the CFH promoter containing the STAT’s core sequences were: Probe 1 (−950 to −927, 5′-ATGCACTTACCAGAAATGTGGATT-3′); Probe 2 (+161 to +184, 5′-AAACAGAATTCTTGGAAGAGGAGA-3′). These DNA probes were end-labeled with 32P-ATP using T4 polynucleotide kinase (Promega, USA). The STAT4 protein was synthesized from STAT4 DNA by using the TNT coupled reticulocyte lysate system (Promega). 

### 4.9. ChIP Assays

ChIP assays were performed as described previously [22]. A549 cells were treated with formaldehyde to crosslink chromatin and nuclear proteins. After sonication, 2 μg of STAT4 antibody was added to precipitate the sheared chromatin. The DNA was purified and used for PCR with the following primers [22]:

F:5′-AAACTCGAGCCAAATTCATCAAGCACTGCATTCTTGGCA-3′;
R:5′-AAAAAGCTTGGATCTTTTAAGAGGACATTTACCAGCTAA-3′.

### 4.10. Bisulfite Conversion PCR and Sequencing

Genomic DNA was isolated from L132 and A549 cell lines and each 1-μg aliquot of genomic DNA was subjected to bisulfite conversion using an EZ DNA Methylation-Lightning Kit according to the manufacturer’s protocols (Zymo Research Corporation, USA). The methylation status of the regulatory regions of three target regulator genes—SOCS1, SOCS3, and STAT4—was determined by the bisulfite conversion–polymerase chain reaction (BS-PCR) using the primer sets and conditions listed in Supplementary Table S2 [32]. Amplicons were gel-extracted using a QIAquick Gel Extraction Kit (Qiagen, Hilden, Germany) and cloned into pGEM T-vector. Plasmid DNA was isolated from up to 25 colonies using the Plasmid Miniprep Kit (iNtRON) and sequencing was performed by Macrogen (Seoul, Korea).

### 4.11. Statistical Analysis 

Data are expressed as the mean ± SD. All statistical analyses were performed using SPSS 19.0 software (IBM SPSS Statistics). For *p*-value calculations, the Mann–Whitney *U* test was applied. A *p*-value < 0.05 was considered significant. 

## 5. Conclusions

In conclusion, our results highlighted that CFH overexpression in lung cancer is due to the demethylation of the STAT4 regulatory region, resulting in its over expression, which may be activated by the JAK/STAT pathway negatively controlled by SOCS-1 proteins (Figure 6). However, to better understand the regulatory mechanism of CFH expression in lung cancer, it would be insightful to investigate its regulation within the complement system because many other complement regulator proteins, such as FHL-1 and FHRs, may be able to influence CFH expression in different levels and pathways. Furthermore, it would be interesting to compare the complement attack susceptibility of cells secreting FH with cells that do not.

## Supplementary Materials

The following are available online at https://www.mdpi.com/2072-6694/11/4/471/s1. Figure S1: mRNA expression of complement regulators, Figure S2: Methylated CpG in SOCSs, Table S1: PCR primers of target genes list, Table S2: Primer set for bisulfite conversion PCR.
