# Supplementary material for: Upregulation of Complement Factor H by SOCS-1/3–STAT4 in Lung Cancer
Source: Cancers (Basel). 2019 Apr 3;11(4):471. doi: 10.3390/cancers11040471 (PMC6520728; doi:10.3390/cancers11040471)
Supplement: Supplementary file 1 [file cancers-11-00471-s001.zip › supplementary figures.pptx]

## Slide 1
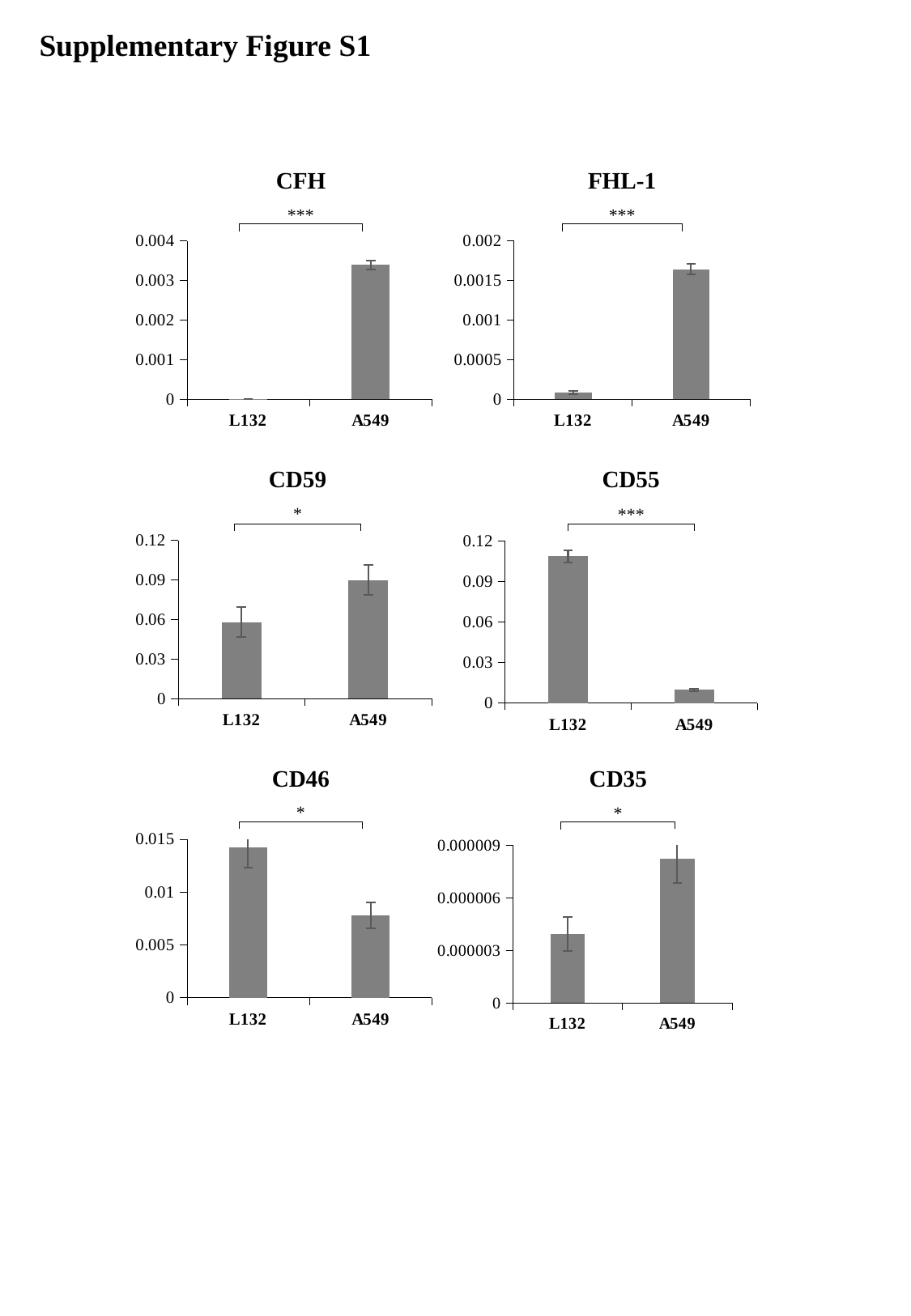

Supplementary Figure S1
CFH
FHL-1
***
***
### Chart
| Category | CFH |
|---|---|
| L132 | 1.0158848993043847e-06 |
| A549 | 0.0033947350558900836 |
### Chart
| Category | FHL-1 |
|---|---|
| L132 | 8.84158716063907e-05 |
| A549 | 0.0016437058890136783 |CD59
CD55
*
***
### Chart
| Category | CD59 |
|---|---|
| L132 | 0.058054637567871485 |
| A549 | 0.09000764117415568 |
### Chart
| Category | CD55 |
|---|---|
| L132 | 0.10840879034952065 |
| A549 | 0.009728133275377781 |CD46
CD35
*
*
### Chart
| Category | CD46 |
|---|---|
| L132 | 0.014259152515739863 |
| A549 | 0.007779097252378145 |
### Chart
| Category | CD35 |
|---|---|
| L132 | 3.939839097292466e-06 |
| A549 | 8.231691376392775e-06 |

## Slide 2
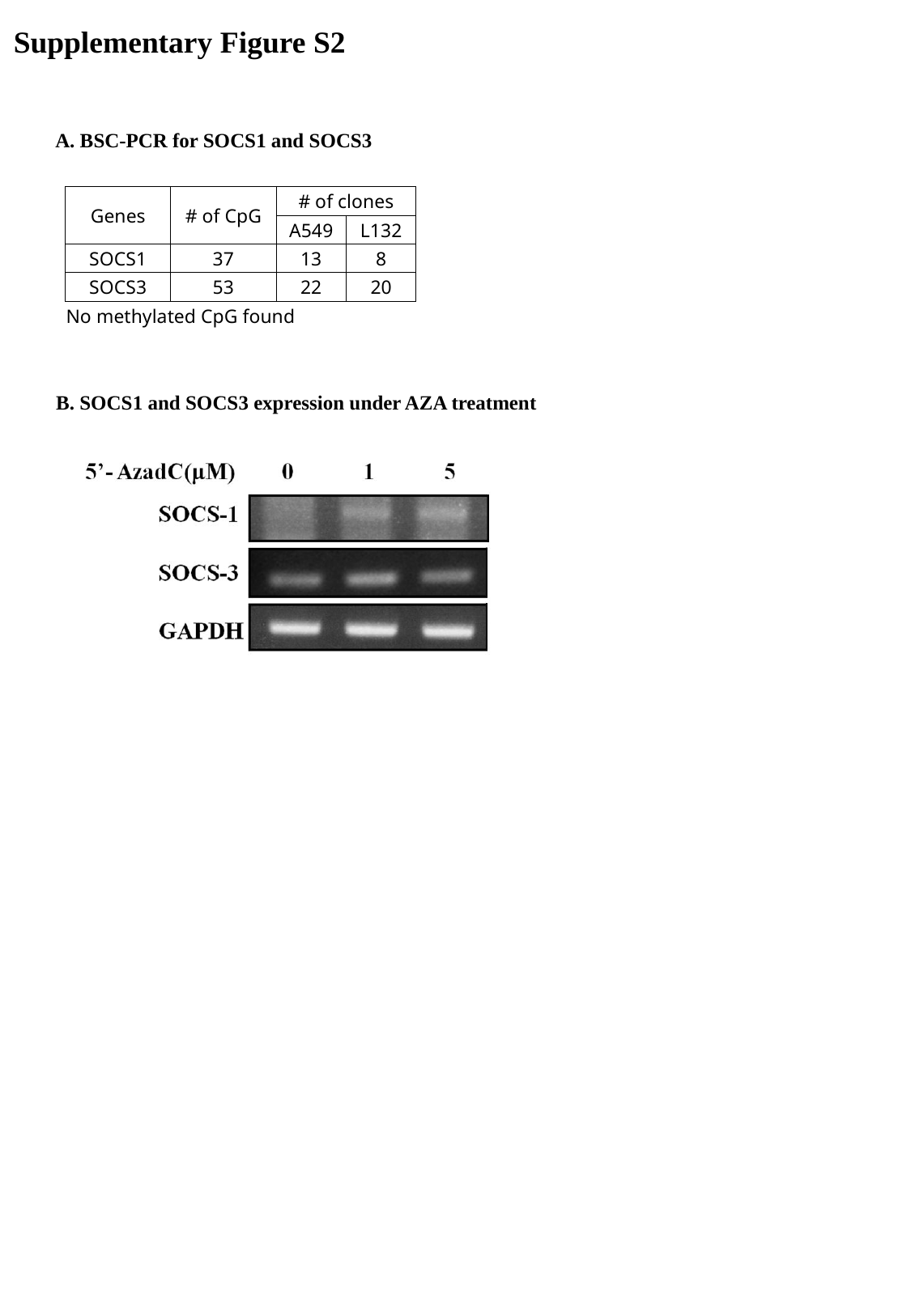

Supplementary Figure S2
A. BSC-PCR for SOCS1 and SOCS3
| Genes | # of CpG | # of clones | |
| --- | --- | --- | --- |
| | | A549 | L132 |
| SOCS1 | 37 | 13 | 8 |
| SOCS3 | 53 | 22 | 20 |
| No methylated CpG found | | | |
B. SOCS1 and SOCS3 expression under AZA treatment
